# Simultaneous administration of imipenem/cilastatin/relebactam with selected intravenous antimicrobials, a stewardship approach

**DOI:** 10.1371/journal.pone.0233335

**Published:** 2020-05-18

**Authors:** Islam M. Ghazi, Wasim S. El Nekidy, Regan Asay, Paul Fingmori, Anthony Knarr, Mina Awad

**Affiliations:** 1 Philadelphia College of Pharmacy, University of the Sciences, Philadelphia, PA, United States of America; 2 Cleveland Clinic Abu Dhabi, UAE and Cleveland Clinic Lerner College of Medicine, Abu Dhabi, United Arab Emirates; University of Lincoln, UNITED KINGDOM

## Abstract

Imipenem/cilastatin/relebactam is a β-lactam/β-lactamase inhibitor that has been recently FDA approved for complicated intra-abdominal and urinary tract infections under the brand name Recarbrio®. It has activity against imipenem non-susceptible *Pseudomonas* species as well as KPC-producing *Enterobacteriaceae*. Optimization of PK/PD of antimicrobials particularly in critically-ill patients is essential, but unfortunately, is hindered by separate administration that requires significant resources. The objective of the study is to investigate the compatibility of Y-site administration of imipenem/cilastatin/relebactam with a wide range of antimicrobials. After admixture, physical characteristics, pH changes and turbidity were measured for each 2-drug combination at a time. With the exception of amphotericin B deoxycholate, and posaconazole, imipenem/cilastatin/relebactam was compatible with a variety of antimicrobial agents. The compatibility profile described, will facilitate incorporation into hospital protocols, contribute to therapy optimization and guide clinicians to avoid successive administration, consequently resulting in reduction of total infusion time, optimization of PK/PD, economizing nursing time and cost containment.

## Introduction

Fervent efforts are underway to develop novel antibiotics to subdue growing bacterial resistance. Imipenem/cilastatin/relebactam is a β-lactam/β-lactamase inhibitor with activity against imipenem non-susceptible *Pseudomonas* species as well as KPC-producing *Enterobacteriaceae* [1,2]. Recently, it was FDA approved for complicated intra-abdominal (cIA)and urinary tract infections (cUTI) under the brand name Recarbrio^®^. A recently published phase III study, The RESTORE IMI-1 demonstrated safety and tolerability in patients infected with carbapenem non-susceptible bacterial pathogens [3]. A press release [4] announced that the RESTORE IMI-2, a phase 3 study in hospital-acquired and ventilator associated bacterial pneumonia has met the primary endpoint.

Optimization of antimicrobials based on pharmacokinetics/pharmacodynamics (PK/PD) is critical in hospitalized patients and especially in critically ill ones. Intravenous (IV) antimicrobials should be infused through separate peripheral lines, different central line lumens, or staggered based on priority, infusion time, and dosing intervals to attain the PK/PD outcomes [5]. The separation process requires extensive attention, critical thinking, and could consume a significant amount of healthcare providers’ time.

The availability of y-site compatibility data between potentially co-administered intravenous antimicrobials would contribute to direct cost savings, reduce nursing time, and enhance the clinical utility in health care settings. We sought to assess the physical compatibility of imipenem/cilastatin/relebactam, when diluted for infusion in suitable diluent; sodium chloride 0.9% (NS) and dextrose 5% in water (D5W) solutions for injection during simulated Y-site administration with common parenteral IV antimicrobials to assist clinicians and stewardship programs with drug administration protocols.

## Methods

### Study test agents

Imipenem/cilastatin/relebactam drug product vials for injection was supplied by Merck & Co Inc, (Kenilworth, NJ, USA) for use during the *in vitro* experiments. Intravenous antimicrobials tested for compatibility with imipenem/cilastatin/relebactam were purchased from a whole sale supplier (McKesson, USA), or from the pharmacy department at Cooper University Health, NJ, USA. The compatibility tests were performed using a two-drug combination at a time, in the intravenous fluid solutions in which both drugs are recommended for administration by their respective manufacturers (either NS or D5W or both separately). The studied drugs were reconstituted for experimentation at the standard concentrations for clinical use for intravenous admixtures, at the upper limit of the concentration range as recommended by the respective manufacturer, or as commercially available at the time of the study. The number of antimicrobials in this study were determined based on: the commonly used ones in practice and the expected co-administration with imipenem/cilastatin/relebactam in consultation with the sponsor. Drug particulars; manufacturer, lot number and expiry date were recorded for each drug.

### Reconstitution and preparation

Imipenem/cilastatin/relebactam vials were reconstituted and diluted to the final concentration 5mg/mL as recommended by the manufacturer. The study intravenous drugs were compounded in appropriate diluent(s) or undiluted as outlined in each of the drug’s monograph. The resulting solutions were prepared immediately prior to the experimentation and refrigerated at (2 to 8°C) and/or light protected by wrapping in aluminum foil, if recommended in the drug monograph.

### Simulated Y-site administration

A 5mL sample of imipenem/cilastatin/relebactam at the desired concentration (5mg/mL) was combined with a 5 mL sample of each of the tested intravenous drug solutions in a colorless, 15-mL, borosilicate glass, screw-cap culture tube with polypropylene caps to simulate the inline mixing through a Y-injection site in a 1:1 ratio [6, 7]. Each of the sample solutions passed through a 0.22-μm filter syringe as it is introduced into the culture tube. Each combination was prepared in duplicate, reversing the order of component addition between the two samples so that there are four vials containing the mixture (n = 2 for each order prepared).

### Visual inspection

Visual inspection and turbidity assessments were conducted as detailed in the following text. After mixing, samples were visually inspected with an unaided eye under normal fluorescent light against black and white backgrounds at 0, 15, 60- and 120-minute intervals. Samples were then stored at room temperature (range 20-25°C), under constant fluorescent light throughout the mixing and until the end of the experiment (2 hours). Combinations were further inspected by passing a direct laser pointer beam through the borosilicate culture tubes to garner a Tyndall effect should any particulate matter forms. Incompatibility was also observed if there was any visible particulate matter, haze, or color changes. For drugs that are typically administered by intravenous bolus or rapid infusion, testing was done at 0, 15, 60 and 120 -minutes intervals only.

### Turbidity assessments

Sample turbidity was measured through laboratory-grade turbidimeter (Hach, USA) in 10 mL borosilicate glass tubes according to the operating instructions. The original storage tube was gently inverted three times, and then a 5 mL from each vial above was transferred to the 10 mL tube for turbidity assessment. Determinations were made for samples at 0, 15, 60 and 120 -minute intervals after mixing. Physical incompatibility was defined as a change in measured turbidity of ≥ 0.5 nephelometric turbidity units (NTU) [8, 9]. Duplicate test combinations were prepared for evaluation at the same time point of as used for the other drugs (0, 15, 60 and 120 -minutes intervals after mixing), reversing the order of drug addition into each pair of test tubes. Samples were stored at room temperature (20 to 25 C) in normal laboratory fluorescent light, as previously described [10].

### Assessment of pH

At the same time points described above (0, 15 min, 60 and 120 -minute intervals after mixing) and prior to mixing (imipenem/cilastatin/relebactam control), sample pH was determined in triplicate for each mixture in the 10 mL borosilicate tube with a laboratory grade pH meter (Fischer Scientific, USA). The wide pH changes were utilized to confirm any physical incompatibilities that may have been observed via Tyndall effect and turbidity measurement. Additionally, if the pH of a mixture that fell outside of the range which is considered safe for intravenous administration (pH: 4–9), was reported as such.

### Experimental controls

Control solutions (negative) were prepared in borosilicate glass tubes with 5 mL of imipenem/cilastatin/relebactam solution and passed through a 0.22-μm filter in a filter syringe, while it is introduced into the culture tube. An additional 5 mL of the diluting solution was added to simulate test sample preparation in 1:1 ratio with reversing the order of addition. Control solutions for phenytoin and haloperidol (D5W only) were prepared as explained above to establish a positive control.

## Results

A collection of antimicrobial agents was tested for Y- site compatibility with imipenem/cilastatin/relebactam. (Table 1) Twenty five antimicrobials were tested in normal saline and 22 antimicrobials were tested in D5W, as diluent solutions depending on individual product-diluent compatibility. After admixture with imipenem/cilastatin/relebactam, tested admixed solutions were clear, colorless, and with no demonstrable haze, except for the following drugs; amphotericin B, doxycycline, levofloxacin, tigecycline (yellow color), and posaconazole (white, hazy and turbid solution). Tyndall effect was observed only in the posaconazole admixed solution. Observations were the same in both diluents. (Table 2)

**Table 1:** Imipenem/cilastatin/relebactam compatibility with tested antimicrobials.

Drug	Concentration* (mg/mL)	NS	D5W
Acyclovir	7	Comp	Comp
Amikacin sulfate	10	Comp	Comp
Amphotericin B deoxycholate	0.25	N/A	Incomp
Ampicillin/sulbactam	20+10	Comp	N/A
Anidulafungin	0.77	Comp	N/A
Azithromycin	2	Comp	Comp
Aztreonam	20	Comp	Comp
Caspofungin	0.5	Comp	N/A
Ceftolozane/tazobactam	10+5	Comp	Comp
Clindamycin	12	Comp	Comp
Colistimethate sodium	4.5	Comp	Comp
Doxycycline hyclate	1	Comp	Comp
Fluconazole	2	Comp	Comp
Gentamicin sulfate	5	Comp	Comp
Isavuconazole	1.5	Comp	Comp
Levofloxacin	5	Comp	Comp
Linezolid	2	Comp	Comp
Metronidazole	5	Comp	Comp
Micafungin sodium	4	Comp	Comp
Moxifloxacin	1.6	Comp	Comp
Plazomicin	24	Comp	Comp
Polymyxin B	1667U/mL	N/A	Comp
Posaconazole	2	Incomp	Incomp
Tedizolid	0.8	Comp	N/A
Tigecycline	1	Comp	Comp
Tobramycin sulfate	5	Comp	Comp
Vancomycin	10	Comp	Comp
Voriconazole	5	Comp	Comp
Imipenem/cilastatin/relebactam^a^	5	Comp	Comp
Haloperidol^b^	5	Incomp	Incomp
Phenytoin^b^	5	Incomp	Incomp

*Concentration expressed in terms of the commercially available form in mg/mL unless otherwise specified

Negative control ^a^

Positive control ^b^

N/A; not applicable, Comp; compatible, Incomp; incompatible

**Table 2:** Summary of physical characteristics of tested antimicrobials in combination with imipenem/cilastatin/relebactam (same observations in both NS and D5W).

Drug	Black background		White background		Tyndall
	Clarity	Haze	Clarity	color	
Acyclovir	Clear	None	Clear	Colorless	Negative
Amikacin sulfate	Clear	None	Clear	Colorless	Negative
Amphotericin B deoxycholate	Clear	None	Clear	Light Yellow	Negative
Ampicillin/sulbactam	Clear	None	Clear	Colorless	Negative
Anidulafungin	Clear	None	Clear	Colorless	Negative
Azithromycin	Clear	None	Clear	Colorless	Negative
Aztreonam	Clear	None	Clear	Colorless	Negative
Caspofungin	Clear	None	Clear	Colorless	Negative
Ceftolozane/tazobactam	Clear	None	Clear	Colorless	Negative
Clindamycin	Clear	None	Clear	Colorless	Negative
Colistimethate sodium	Clear	None	Clear	Colorless	Negative
Doxycycline hyclate	Clear	None	Clear	Light Yellow	Negative
Fluconazole	Clear	None	Clear	Colorless	Negative
Gentamicin sulfate	Clear	None	Clear	Colorless	Negative
Isavuconazole	Clear	None	Clear	Colorless	Negative
Levofloxacin	Clear	None	Clear	Light Yellow	Negative
Linezolid	Clear	None	Clear	Colorless	Negative
Metronidazole	Clear	None	Clear	Colorless	Negative
Micafungin sodium	Clear	None	Clear	Colorless	Negative
Plazomicin	Clear	None	Clear	Colorless	Negative
Polymyxin B	Clear	None	Clear	Colorless	Negative
Posaconazole	Turbid	Hazy	Turbid	White	Positive
Tedizolid	Clear	None	Clear	Colorless	Negative
Tigecycline	Clear	None	Clear	Yellow	Negative
Tobramycin sulfate	Clear	None	Clear	Colorless	Negative
Vancomycin	Clear	None	Clear	Colorless	Negative
Voriconazole	Clear	None	Clear	Colorless	Negative
Imipenem/cilastatin/relebactam*	Clear	None	Clear	Colorless	Negative
Haloperidol**	Clear	None	Clear	Light yellow	Negative
Phenytoin**	Cloudy	Hazy	Cloudy	Yellow	Positive

Negative control *

Positive control **

For pH measurements, (Tables 3 and 4), all study solutions were within the range of 4 to 9, with 2 notable exceptions, colistimethate sodium and gentamicin solutions which had some readings slightly below pH value of 4. As per study definition (turbidity increase of ≥ 0.5 NTU), amphoterin B, and posaconazole were found to be incompatible with imipenem/cilastatin/relebactam for simultaneous administration. The admixture of imipenem/cilastatin/relebactam with blank solutions manifested no sign of incompatibility as a negative control while, phenytoin in both NS and D5W and haloperidol in D5W were incompatible yielding readings of ≥ 0.5 NTU and visible development of turbidity as positive controls.

**Table 3:** Summary of pH changes in the tested antimicrobials over 120 minutes in normal saline.

Drug	NS			
	pH immediately after mixing (0 min)	pH change 15 min	pH change 60 min	pH change 120 min
Acyclovir	6.80	-0.07	-0.01	0.03
Amikacin sulfate	6.80	-0.38	-0.13	0.07
Ampicillin/sulbactam	5.97	0.06	-0.30	-0.67
Anidulafungin	6.70	-0.12	-0.08	-0.05
Azithromycin	5.08	0.26	0.32	0.21
Aztreonam	6.17	0.32	0.10	0.08
Caspofungin	6.74	-0.13	-0.14	-0.09
Ceftolozane/tazobactam	6.75	-0.04	-0.02	0.03
Clindamycin	6.84	-0.32	-0.32	-0.31
Colistimethate sodium	6.99	-0.21	-0.23	-0.26
Doxycycline hyclate	7.89	0.11	0.32	0.17
Fluconazole	6.97	-0.27	-0.33	-0.23
Gentamicin sulfate	5.18	0.08	-0.08	-0.20
Isavuconazole	6.69	-0.28	-0.08	0.02
Levofloxacin	5.08	-0.02	0.01	-0.05
Linezolid	6.44	-0.78	-0.71	-0.82
Metronidazole	5.89	0.06	0.03	0.00
Micafungin sodium	6.96	-0.05	0.57	0.01
Plazomicin	6.53	-0.31	-0.30	-0.29
Posaconazole	6.90	-0.14	-0.22	-0.37
Tedizolid	6.17	-0.23	-0.22	-0.32
Tigecycline	5.98	0.13	0.08	-0.35
Tobramycin sulfate	6.30	-0.42	-0.21	0.25
Vancomycin	6.75	-0.02	-0.02	0.01
Voriconazole	7.94	0.14	0.35	0.19
Imipenem/cilastatin/relebactam*	6.84	0.03	0.1	0.1
Phenytoin**	6.75	0.05	0.12	0.06

Negative control *

Positive control **

**Table 4:** Summary of pH changes in the tested antimicrobials over 120 minutes in dextrose 5% in water.

Drug	D5W			
	pH immediately after mixing (0 min)	pH change 15 min	pH change 60 min	pH change 120 min
Acyclovir	6.57	0.16	0.27	0.78
Amikacin sulfate	6.55	0.20	0.34	0.70
Amphotericin B deoxycholate	6.80	-0.14	-0.10	-0.10
Azithromycin	4.81	0.56	0.49	1.29
Aztreonam	6.09	0.10	0.51	0.39
Ceftolozane/tazobactam	6.69	-0.04	0.03	-0.12
Clindamycin	7.20	0.09	0.37	0.17
Colistimethate sodium	4.94	0.97	0.52	1.68
Doxycycline hyclate	7.62	-0.06	0.17	0.23
Fluconazole	5.93	-0.55	-0.55	-0.33
Gentamicin sulfate	4.91	0.84	0.51	1.56
Isavuconazole	6.44	-0.15	0.40	0.50
Levofloxacin	5.09	0.05	0.58	0.77
Linezolid	5.87	-0.53	-0.49	-0.45
Metronidazole	4.99	-0.06	-0.09	0.07
Micafungin sodium	6.52	-0.14	-0.12	-0.70
Polymyxin B	5.90	-0.03	0.18	0.23
Posaconazole	6.52	-0.35	-0.38	-0.42
Tigecycline	5.92	-0.08	0.16	0.18
Tobramycin sulfate	6.50	0.19	0.26	0.83
Vancomycin	7.40	0.22	0.36	0.34
Voriconazole	7.42	-0.22	0.10	0.20
Imipenem/cilastatin/relebactam*	6.62	0.21	0.23	0.17
Haloperidol**	6.32	0.42	0.5	0.61
Phenytoin**	6.3	0.51	0.48	0.6

Negative control *

Positive control **

## Discussion

When tested for y-site administration with a variety of antimicrobial agents comprising a wide spectrum of activity, imipenem/cilastatin/relebactam was incompatible with amphotericin B and posaconazole. Alternatively, the drug was compatible with other antifungals (anidulafungin, micafungin, caspofungin and fluconazole). Additionally, the drug was compatible with several antibacterial agents covering a broad spectrum of bacteria including, both Gram-positive and negative bacteria.

In comparison with the parent compound imipenem/cilastatin, Trissel’s (11) reported amphotericin B, ampicillin/sulbactam, azithromycin, aztreonam and fluconazole were reported to be of uncertain compatibility for various reasons. Ampicillin is known to be unstable in dextrose containing solutions, azithromycin in NS formed white microcrystalline precipitate on a 0.8-micron filter when examined microscopically, aztreonam in D5W was compatible at 40 mg/mL and incompatible at 80 mg/mL and fluconazole 2mg/mL precipitated immediately when mixed undiluted [11]. However, in our study amphotericin B was found to be incompatible and the above listed antimicrobials were compatible. The dissimilarity could be attributed to different definitions of incompatibility, drug concentrations used in the experiment or discrepant findings of several studies reported to Trissel’s [11].

It is noteworthy that, since several antimicrobials were previously reported to be incompatible with imipenem/cilastatin [11] (amphotericin B cholesteryl, amphotericin B lipid complex, amphotericin B liposome, ceftriaxone, daptomycin, ganciclovir, minocycline, quinupristin and dalfopristin, sulfamethoxazole-trimethoprim and temocillin), they were excluded from the current study based on the expected incompatibility with imipenem/cilastatin/relebactam.

A recent study which evaluated simultaneous Y-site administration of similar carbapenem/carbapenemase inhibitor combination (meropenem/vaborbactam), found the compound to be compatible only with one antifungal, micafungin, while it was incompatible with anidulafungin, caspofungin, isavuconazole, and not tested against amphotericin B, fluconazole, posaconazole, voriconazole. Imipenem/cilastatin/relebactam was compatible with several antifungal agents as listed above. Regarding antibiotics with MRSA activity, meropenem/vaborbactam was compatible with linezolid, tedizolid and vancomycin, (incompatible with daptomycin and ceftaroline), comparably, imipenem/cilastatin/relebactam was compatible with linezolid, tedizolid and vancomycin but not tested with neither ceftaroline nor daptomycin. Both compounds were compatible with aminoglycosides (amikacin, gentamicin and tobramycin) in case synergy is required. Finally, imipenem/cilastatin/relebactam was compatible with acyclovir, while meropenem/vaborbactam was not tested with any antiviral agents [12].

As a relevant point of comparison, while imipenem/cilastatin/relebactam does not need prolonged infusion time to achieve target *f*T>MIC (30-min infusion every 6 hours), meropenem/vaborbactam is recommended for 3-hour infusion every 8 hours. In case of unknown compatibility or incompatibility antimicrobial that requires prolonged or continuous infusion, a robust logistic plan must be in place to accommodate the separate administration of the two drugs around the clock.

Antimicrobial stewardship programs aim to provide patients with the most effective treatment, while reducing costs. Simultaneous administration of antibiotics allows; the use of antibiotic combinations for serious infections without the need for dedicated lines, reducing the total time needed for infusion while allowing a window for PK/PD manipulation and shortening nursing time required for administration/observation. The availability of drug compatibility data guides the design of stewardship protocols on which antibiotic combinations can be administered via shared lines and eliminates the need for successive administration, thus producing time and cost savings.

A limitation of this study is that the physical compatibility profile is described for only 120 minutes and possibility of incompatibility beyond that time frame precludes co-administration of imipenem/cilastatin/relebactam with tested intravenous antimicrobials for longer period of time.

## Conclusion

The compatibility data generated from this study, will aid clinicians in overcoming difficulties pertinent to successive administration in terms of reducing total infusion time, optimizing PK/PD exposure, by allowing prolonged/extended infusion to be accommodated on top of economizing nursing time, and cost.
