# Correction: Simultaneous administration of imipenem/cilastatin/relebactam with selected intravenous antimicrobials, a stewardship approach

**DOI:** 10.1371/journal.pone.0235405

**Published:** 2020-06-24

**Authors:** Islam M. Ghazi, Wasim S. El Nekidy, Regan Asay, Paul Fimognari, Anthony Knarr, Mina Awad

The fourth author's name is spelled incorrectly. The correct name is: Paul Fimognari. The correct citation is: Ghazi IM, El Nekidy WS, Asay R, Fimognari P, Knarr A, Awad M (2020) Simultaneous administration of imipenem/cilastatin/relebactam with selected intravenous antimicrobials, a stewardship approach. PLoS ONE 15(5): e0233335. https://doi.org/10.1371/journal.pone.0233335
